# Lineage-Specific Analysis of Syk Function in Autoantibody-Induced Arthritis

**DOI:** 10.3389/fimmu.2018.00555

**Published:** 2018-03-19

**Authors:** Tamás Németh, Krisztina Futosi, Kata Szilveszter, Olivér Vilinovszki, Levente Kiss-Pápai, Attila Mócsai

**Affiliations:** ^1^Department of Physiology, Semmelweis University School of Medicine, Budapest, Hungary; ^2^MTA-SE “Lendület” Inflammation Physiology Research Group of the Hungarian Academy of Sciences and Semmelweis University, Budapest, Hungary

**Keywords:** Syk, arthritis, neutrophils, platelets, mast cells

## Abstract

Autoantibody production and autoantibody-mediated inflammation are hallmarks of a number of autoimmune diseases. The K/BxN serum-transfer arthritis is one of the most widely used models of the effector phase of autoantibody-induced pathology. Several hematopoietic lineages including neutrophils, platelets, and mast cells have been proposed to contribute to inflammation and tissue damage in this model. We have previously shown that the Syk tyrosine kinase is critically involved in the development in K/BxN serum-transfer arthritis and bone marrow chimeric experiments indicated that Syk is likely involved in one or more hematopoietic lineages during the disease course. The aim of the present study was to further define the lineage(s) in which Syk expression is required for autoantibody-induced arthritis. To this end, K/BxN serum-transfer arthritis was tested in conditional mutant mice in which Syk was deleted in a lineage-specific manner from neutrophils, platelets, or mast cells. Combination of the MRP8-Cre, PF4-Cre, or Mcpt5-Cre transgene with floxed Syk alleles allowed efficient and selective deletion of Syk from neutrophils, platelets, or mast cells, respectively. This has also been confirmed by defective Syk-dependent *in vitro* functional responses of the respective cell types. *In vivo* studies revealed nearly complete defect of the development of K/BxN serum-transfer arthritis upon neutrophil-specific deletion of Syk. By contrast, Syk deletion from platelets or mast cells did not affect the development of K/BxN serum-transfer arthritis. Our results indicate that autoantibody-induced arthritis requires Syk expression in neutrophils, whereas, contrary to prior assumptions, Syk expression in platelets or mast cells is dispensable for disease development in this model.

## Introduction

A number of autoimmune diseases, including rheumatoid arthritis, systemic lupus erythematosus, small vessel vasculitis, or pemphigoid diseases, are characterized by production of autoantibodies against various autoantigens of the mammalian body (1). Those autoantibodies are thought to contribute to the autoimmune disease pathogenesis, either directly by engagement of their target autoantigens (activating or function-blocking autoantibodies), or by triggering an inflammatory reaction and concomitant tissue damage caused by the infiltrating inflammatory cells.

The K/BxN serum-transfer arthritis is one of the most widely used mouse model of autoantibody-induced tissue damage. This model is initiated by systemic injection of serum from so-called K/BxN mice in which the expression of a specific T-cell-receptor transgene on an autoimmunity-prone genetic background leads to the generation of high titers of autoantibodies against the ubiquitously expressed glucose 6-phosphate isomerase enzyme (2–5). Transferring those autoantibodies with the K/BxN serum to naive animals triggers robust inflammation of the distal joints and of other tissues. K/BxN serum-transfer arthritis is triggered by immune complex (IC) deposition and concomitant activation of Fcγ-receptors (5). A number of hematopoietic lineages are thought to be involved in the development of K/BxN serum-transfer arthritis. The role of neutrophils is indicated by the fact that antibody-mediated depletion (6) or genetic deletion (7, 8) of neutrophils prevents arthritis development in this model. Arthritis development was also reduced in mast cell-deficient *Kit*^W/W-v^ mice (9) suggesting an important role of mast cells. In addition, platelets were proposed to be required for the development of K/BxN serum-transfer arthritis by releasing platelet-derived microparticles upon collagen-induced activation in the synovial tissue (10).

Syk is a nonreceptor tyrosine kinase primarily expressed in cells of the hematopoietic lineage (11). It mediates signaling by a number of cell surface receptors including B-cell-receptors (12, 13), Fcγ- and Fcε-receptors (14–18), β_2_ and β_3_ integrins (19–21), C-type lectins (11, 22), and other receptors coupled to immunoreceptor tyrosine-based activation motifs (ITAMs) (11, 23). Given its role in various hematopoietic lineages and signaling downstream of diverse cell surface receptors, Syk is indispensable for a number of *in vivo* processes including B-cell development (12, 13), various inflammatory disease processes (17, 24, 25), antifungal immunity (26), or lymph vessel development (27). Based on its central role in the immune system, Syk has been proposed as a therapeutic target in various autoimmune and inflammatory diseases (11, 28).

We have previously shown that Syk is critically involved in arthritis development in the autoantibody-induced K/BxN serum transfer model (25). Our additional studies indicated that Syk is involved in a pathway downstream of Fc-receptors and Src-family kinases (29) and activates further downstream processes through PLCγ2 (30) and CARD9 (31). However, it is at present incompletely understood in which lineage(s) Syk needs to be expressed for arthritis development in this model. Bone marrow chimeric experiments suggested the role for Syk in one or more hematopoietic lineages (25). Several lines of evidence suggest an important role for Syk in neutrophils (19, 31, 32). An important role for GpVI, an ITAM-coupled collagen receptor on platelets, for the development of K/BxN serum-transfer arthritis (10) suggested a role for Syk in platelets for disease development in this model (33). Finally, the proposed role of mast cells (9, 34) and the critical role for Syk in mast cell activation (14, 18) raised the possibility that Syk expression in mast cells contributes to development of K/BxN serum-transfer arthritis.

The above studies prompted us to perform lineage-specific deletion of Syk from neutrophils, platelets, and mast cells, and to test the effect of those mutations on the development of autoantibody-induced arthritis in the K/BxN serum-transfer model. Our results indicate an important role for Syk expression in neutrophils whereas, contrary to our expectations, Syk expression in platelets or mast cells appears to be dispensable for arthritis development in this model.

## Materials and Methods

### Animals

Mice carrying a deleted *Syk* allele (*Syk*^tm1Tyb^, referred to as *Syk^−^*) (12) were kept in heterozygous form and used to obtain *Syk^−^*^/^*^−^* and control fetuses for fetal liver transplantation (19, 25). Lineage-specific deletion of *Syk* was achieved by crossing MRP8-Cre (35), PF4-Cre (36), or Mcpt5-Cre transgenic mice (37) with animals carrying a floxed Syk allele (*Syk*^tm1.2Tara^, referred to as *Syk*^flox^) (38) to obtain MRP8-Cre^+^*Syk*^flox/flox^, PF4-Cre^+^*Syk*^flox/flox^, and Mcpt5-Cre^+^*Syk*^flox/flox^ mice, referred to as *Syk*^ΔPMN^, *Syk*^ΔPLT^, and *Syk*^ΔMC^ animals, respectively. Mice carrying the KRN T-cell-receptor transgene (2) were maintained in heterozygous form by mating with C57BL/6 mice. All transgenic mice were backcrossed to the C57BL/6 genetic background for at least six generations. Genotyping was performed by allele-specific PCR.

Wild type (WT) control C57BL/6 mice were purchased from Charles River or the Hungarian National Institute of Oncology (Budapest, Hungary). NOD mice, as well as a congenic strain carrying the CD45.1 allele on the C57BL/6 genetic background (B6.SJL-*Ptprc*^a^) were purchased from the Jackson Laboratory.

Mice were kept in individually sterile ventilated cages (Tecniplast) in a conventional facility. All animal experiments were approved by the Animal Experimentation Review Board of the Semmelweis University.

Bone marrow chimeras were generated by intravenous injection of unfractionated bone marrow or fetal liver cells into recipients carrying the CD45.1 allele on the C57BL/6 genetic background, which were lethally irradiated before by 11 Gy from a ^137^Cs source using a Gamma-Service Medical (Leipzig, Germany) D1 irradiator. 4 weeks after transplantation, peripheral blood samples were stained for Ly6G and CD45.2 (Clones 1A8 and 104, respectively; both from BD Biosciences) and analyzed by a BD Biosciences FACSCalibur flow cytometer as previously described (see Figure S1A in Supplementary Material) (29).

### K/BxN Serum-Transfer Arthritis

Mice carrying the KRN T-cell receptor transgene on the C57BL/6 genetic background were mated with NOD mice to obtain transgene-positive (arthritic) K/BxN and transgene-negative (non-arthritic) BxN mice (2, 30). The presence of the transgene was determined by allele-specific PCR and confirmed by phenotypic assessment. Blood was taken by retroorbital bleeding and sera from arthritic and control mice were pooled separately.

Arthritis was induced by a single intraperitoneal injection of 300 μl K/BxN (arthritic) or BxN (control) serum into intact mice or bone marrow chimeras, followed by daily assessment of arthritis severity for 2 weeks as described (30, 31, 39). Visible clinical signs were scored on a 0–10 scale by two investigators blinded for the origin and treatment of the mice. Ankle thickness was measured by a spring-loaded caliper (Kroeplin).

### Isolation and Activation of Neutrophils, Platelets, and Mast Cells

Mouse neutrophils were isolated from the bone marrow of the femurs and tibias of intact mice or chimeras by hypotonic lysis followed by Percoll (GE Healthcare) gradient centrifugation using sterile and endotoxin-free reagents as described (18, 31, 39). Cells were kept at room temperature in Ca^2+^- and Mg^2+^-free medium until use and prewarmed to 37°C prior to activation. Neutrophil assays were performed at 37°C in HBSS supplemented with 20 mM HEPES, pH 7.4. Adhesion-dependent superoxide release by neutrophils was followed by a cytochrome *c* reduction test from 100 μl aliquots of 4 × 10^6^/ml cells plated on fibrinogen (Calbiochem) coated surfaces in the presence of 50 ng/ml murine TNF-α (PeproTech) as described (39).

Platelets were isolated from peripheral blood by mild centrifugation in the presence of heparin. For an *in vitro* aggregation assay (40), platelets were divided into two groups, one labeled with an anti-CD9-PE (Clone EM-04; Abcam) and the other one with an anti-CD9-APC (Clone eBioKMC8; eBioscience) antibody. The two differently labeled groups were mixed in equal volumes and were activated by 50 ng/ml Convulxin (Enzo Life Sciences) at 37°C while shaking at 700 rpm for 5 min. The reaction was stopped by BD FACS Lysing Solution (BD Biosciences). The samples were analyzed by flow cytometry, where platelets were identified according to their forward and side scatter characteristics. Aggregation was determined as the percentage of CD9-PE/CD9-APC double positive events (40).

Mast cells were cultured from the bone marrow in the presence of 5 ng/ml murine IL-3 and 20 ng/ml stem cell factor (both from PeproTech). The purity of the cultures was tested by an anti-FcεR antibody (Clone MAR-1; eBioscience) by flow cytometry. For *in vitro* activation, mast cells were first incubated with an anti-dinitrophenyl (DNP) IgE antibody (Clone SPE-7) at a final concentration of 0.5 μg/ml overnight at 37°C on fetal bovine serum (FBS)-coated plates, followed by the crosslinking of Fcε receptors by the addition of 100 ng/ml DNP-human serum albumin to the cell suspensions (both reagents from Sigma-Aldrich). After 30 min, the cells were washed and mast cells were kept in Dulbecco’s Modified Eagle’s Medium (DMEM; Sigma-Aldrich) overnight at 37°C on FBS-coated plates. The release of the inflammatory mediator MIP-1α was tested from the cell-free supernatants by a commercial ELISA kit (R&D Systems) according to the manufacturer’s instructions. The absence of Syk did not have a major effect on neutrophil, platelet, or mast cell development and numbers (data not shown).

### Biochemical Studies

For analysis of protein contents, neutrophils, platelets, and mast cells were lysed in 100 mM NaCl, 30 mM Na-HEPES (pH 7.4), 20 mM NaF, 1 mM Na-EGTA, 1% Triton X-100, 1 mM benzamidine, freshly supplemented with 0.1 U/ml Aprotinin, 1:100 Mammalian Protease Inhibitor Cocktail, 1:100 Phosphatase Inhibitor Cocktail 2, 1 mM PMSF, and 1 mM Na_3_VO_4_ (all from Sigma-Aldrich). After removal of insoluble material, lysates were boiled in sample buffer. Whole cell lysates were run on SDS-PAGE and immunoblotted using antibodies against Syk (Clone N19; Santa Cruz) or β-actin (Clone AC-74; Sigma-Aldrich) followed by peroxidase-labeled secondary antibodies (GE Healthcare). The signal was then developed using the ECL system (GE Healthcare) and exposed to X-ray film.

### Presentation of the Data and Statistical Analysis

Experiments were performed the indicated number of times. Quantitative graphs and kinetic curves show mean and SEM from all independent *in vitro* experiments or from all individual mice from the indicated number of experiments. Statistical analyses were carried out by the STATISTICA software using two-way (factorial) ANOVA, with treatment and genotype being the two independent variables. In case of kinetic assays, area under the curve was used for statistical analysis. P values below 0.05 were considered statistically significant.

## Results

### K/BxN Serum-Transfer Arthritis in Syk^*−*/*−*^ Bone Marrow Chimeras

**Figure 1:**
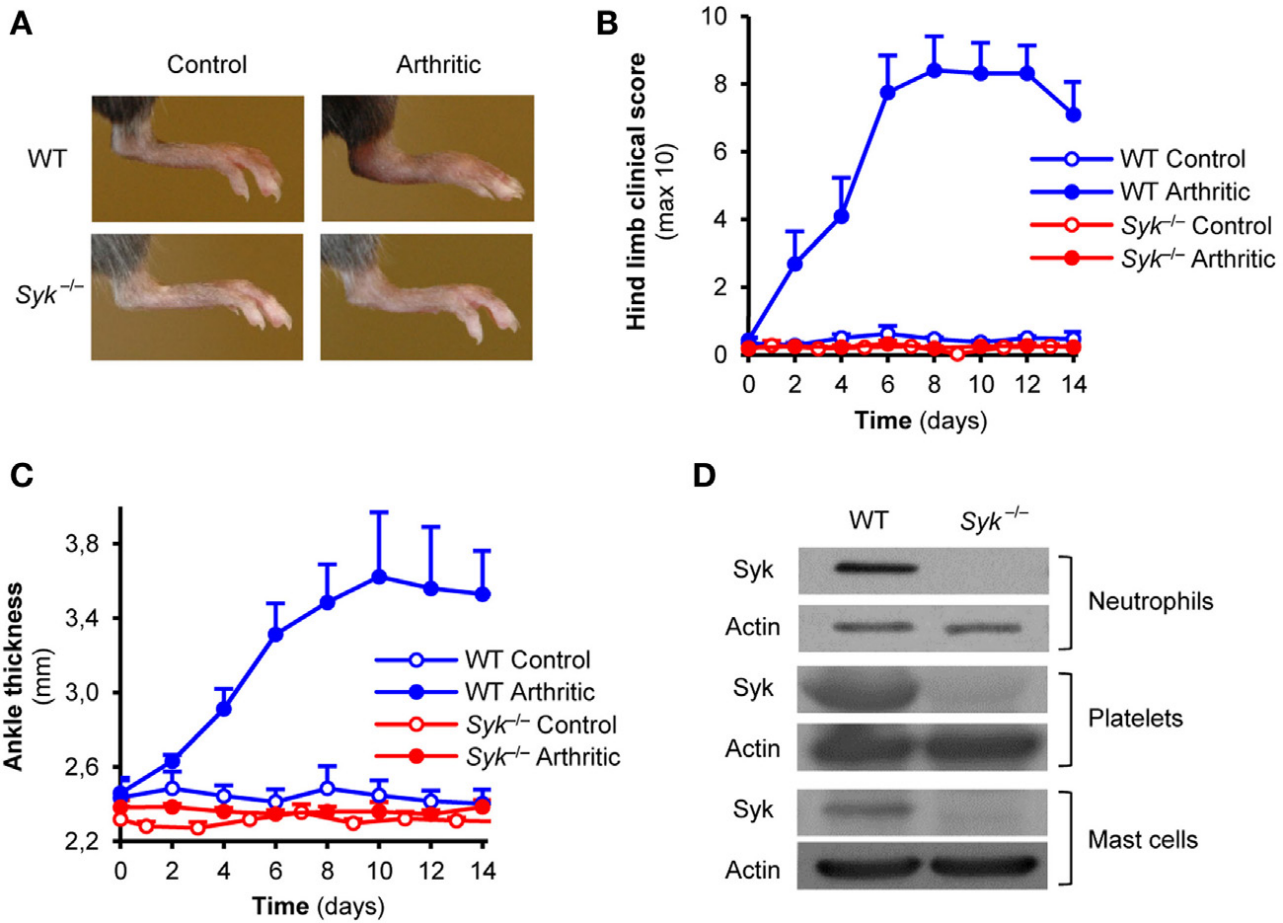
Autoantibody-induced arthritis in Syk-deficient bone marrow chimeras. Wild type (WT) and *Syk^−^*^/^*^−^* bone marrow chimeras were injected with BxN (Control) or K/BxN (Arthritic) serum intraperitonally on day 0. Arthritis development was followed by photographing on day 7 **(A)**, clinical scoring of the hind limbs **(B)**, and ankle thickness measurement **(C)**. Panel **(D)** shows the absence of the Syk tyrosine kinase from whole cell lysates of *Syk^−^*^/^*^−^* neutrophils, platelets, and mast cells. Photos are representative of, and quantitative data show mean and SEM from, four control and four to five arthritic serum-treated individual mice per group from two independent experiments. Western blot images are representative of two to three independent experiments. See the text for actual *p* values.

To test the role of Syk within hematopoietic lineage cells, we generated bone marrow chimeric mice by transplanting *Syk^−^*^/^*^−^* or WT control fetal liver cells into lethally irradiated recipients. As shown in Figure 1A, injection of arthritogenic K/BxN serum into WT control chimeras triggered robust inflammation of the ankle joints whereas no such response could be observed in *Syk^−^*^/^*^−^* bone marrow chimeras which carry Syk-deficient hematopoietic tissues. Quantitative kinetic analysis of the clinical scoring of arthritis (Figure 1B) or the ankle thickness (Figure 1C) revealed that *Syk^−^*^/^*^−^* bone marrow chimeras were completely protected from arthritis development in this model (p = 3 × 10^−6^ and p = 1.3 × 10^−3^, respectively). Those results confirmed our prior studies showing critical role for Syk in the hematopoietic compartment in K/BxN serum-transfer arthritis (25).

### Syk Is Expressed in Neutrophils, Platelets, and Mast Cells

Prior studies suggested a role for neutrophils, platelets, and mast cells in K/BxN serum-transfer arthritis (6–10, 34), as well as the functional role for Syk in those cells (14, 16, 18–20). To confirm the presence of Syk in those lineages and its deletion from *Syk^−^*^/^*^−^* cells, we tested the expression of Syk in primary neutrophils and platelets, or bone marrow-derived mast cells, from WT and *Syk^−^*^/^*^−^* bone marrow chimeras. As shown in Figure 1D, Syk was present in all three cell types derived from WT but not *Syk^−^*^/^*^−^* bone marrow chimeras (see the entire blots in Figures S1B–D in Supplementary Material).

### Efficacy and Specificity of Syk Deletion From Neutrophils, Platelets, and Mast Cells

**Figure 2:**
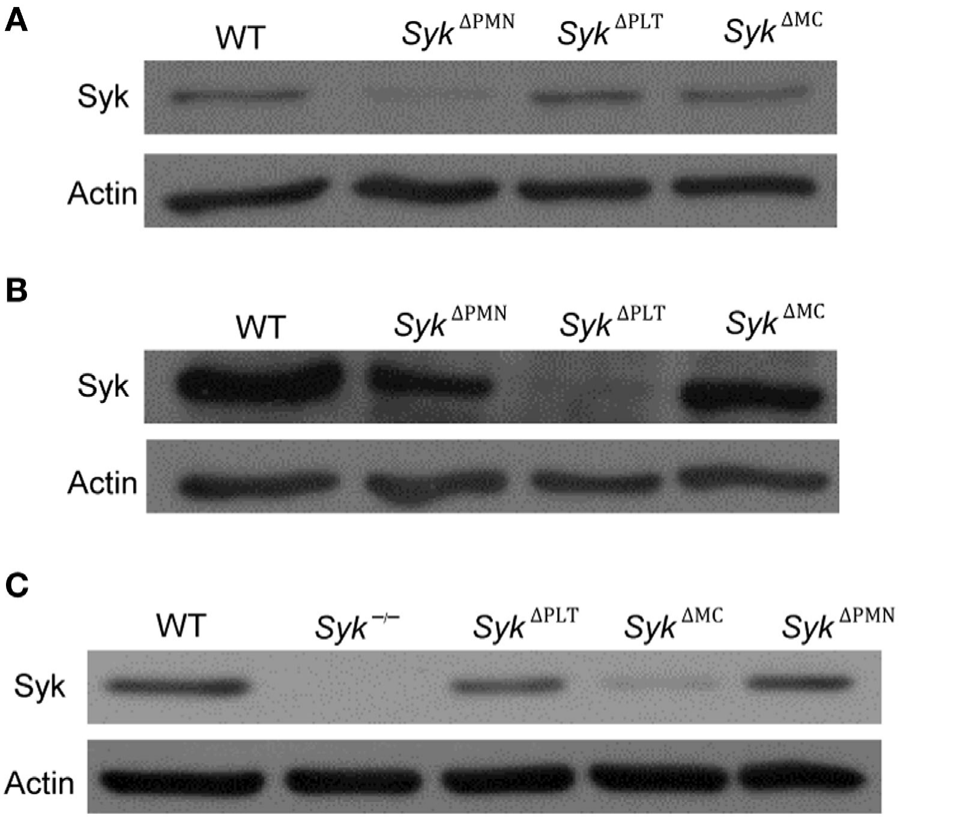
The efficacy and specificity of lineage-specific Syk deletion from different cell types. The efficacy and specificity of lineage-specific deletion was tested by immunoblotting of whole cell lysates of neutrophils **(A)**, platelets **(B)**, and mast cells **(C)** derived from wild type (WT), *Syk^ΔPMN^, Syk^ΔPLT^*, or *Syk^ΔMC^* mice or from *Syk^−^*^/^*^−^* bone marrow chimeras. Blots are representative of three independent experiments.

To test the role of Syk in a linage-specific manner, we turned to Cre-lox-mediated lineage-specific conditional deletion of Syk. To this end, we generated mice carrying the *Syk*^flox/flox^ mutation along with a neutrophil-specific MRP8-Cre (*Syk*^ΔPMN^), the platelet-specific PF4-Cre (*Syk*^ΔPLT^), or the mast cell-specific Mcpt5-Cre (*Syk*^ΔMC^) transgenes. We then isolated neutrophils or platelets, and cultured bone marrow-derived mast cells, from those animals. As shown in Figure 2A, Syk expression was strongly reduced in *Syk*^ΔPMN^, but was not affected in *Syk*^ΔPLT^ or *Syk*^ΔMC^ neutrophils. Similarly, Syk was absent from *Syk*^ΔPLT^ but not from *Syk*^ΔPMN^ or *Syk*^ΔMC^ platelets (Figure 2B). Finally, Syk expression was abrogated in *Syk*^ΔMC^ but not in *Syk*^ΔPMN^ or *Syk*^ΔPLT^ mast cells (Figure 2C; see the entire blots in Figure S2 in Supplementary Material). Those results confirm both the efficacy and the specificity of the *Syk*^ΔPMN^, *Syk*^ΔPLT^, and *Syk*^ΔMC^ mutations.

### Lineage-Specific Deletion of Syk Abrogates Functional Responses of Neutrophils, Platelets, and Mast Cells

**Figure 3:**
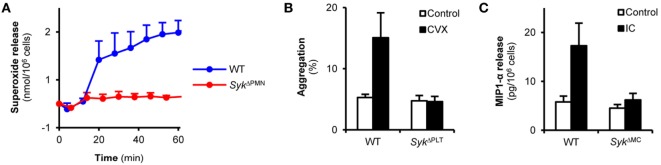
Syk is indispensable for immunoreceptor tyrosine-based activation motif-mediated *in vitro* cellular responses of neutrophils, platelets, and mast cells. **(A)** Wild type (WT) or *Syk*^ΔPMN^ neutrophils were plated on fibrinogen-coated surfaces in the presence of TNF-α and their superoxide release was measured by a cytochrome *c* reduction test. Control data points were subtracted. **(B)** WT or *Syk^ΔPLT^* platelets were isolated from peripheral blood, labeled by two different fluorochrome-conjugated CD9 antibodies and were activated by convulxin (CVX) for 5 min. Aggregation was measured as the percentage of CD9-PE/CD9-APC double positive events. **(C)** WT or *Syk^ΔMC^* bone marrow-derived mast cells were incubated with anti-DNP IgE antibodies followed by an Fcε receptor crosslinking step with DNP-HSA. MIP-1α levels were determined from the cell-free supernatant by an ELISA assay. Kinetic curves and graphs represent mean and SEM from three **(A,C)** or six **(B)** samples from three **(A)** or two **(B,C)** independent experiments. See the text for actual *p* values. DNP, dinitrophenyl; HSA, human serum albumin, IC, immune complex.

To test the functional efficacy of lineage-specific Syk deletion, we also tested supposedly Syk-dependent functional responses of neutrophils, platelets, and mast cells. Superoxide release of neutrophils plated on a fibrinogen surface in the presence of a soluble TNF stimulus is mediated by β_2_ integrins in a supposedly Syk-dependent manner (19). As shown in Figure 3A, this response was nearly completely blocked in neutrophils from *Syk*^ΔPMN^ animals (*p* = 0.02). Convulxin is a snake venom toxin activating the Fc-receptor-related collagen receptor GpVI on platelets in a Syk-dependent manner (40, 41). As shown in Figure 3B, convulxin induced aggregation of WT but not *Syk*^ΔPLT^ platelets (*p* = 0.03). Crosslinking of IgE bound to the surface of mast cells triggers release of various proinflammatory mediators through Fcε-receptors in a Syk-dependent manner (14). As shown in Figure 3C, MIP-1α release induced by IgE crosslinking of mast cells was abrogated by the *Syk*^ΔMC^ mutation (*p* = 1.3 × 10^−4^). Those results indicate that lineage-specific deletion of Syk from neutrophils, platelets, or mast cells leads to the expected functional consequences in those cells.

### Neutrophil-Specific Deletion of Syk Abrogates Autoantibody-Induced Arthritis

**Figure 4:**
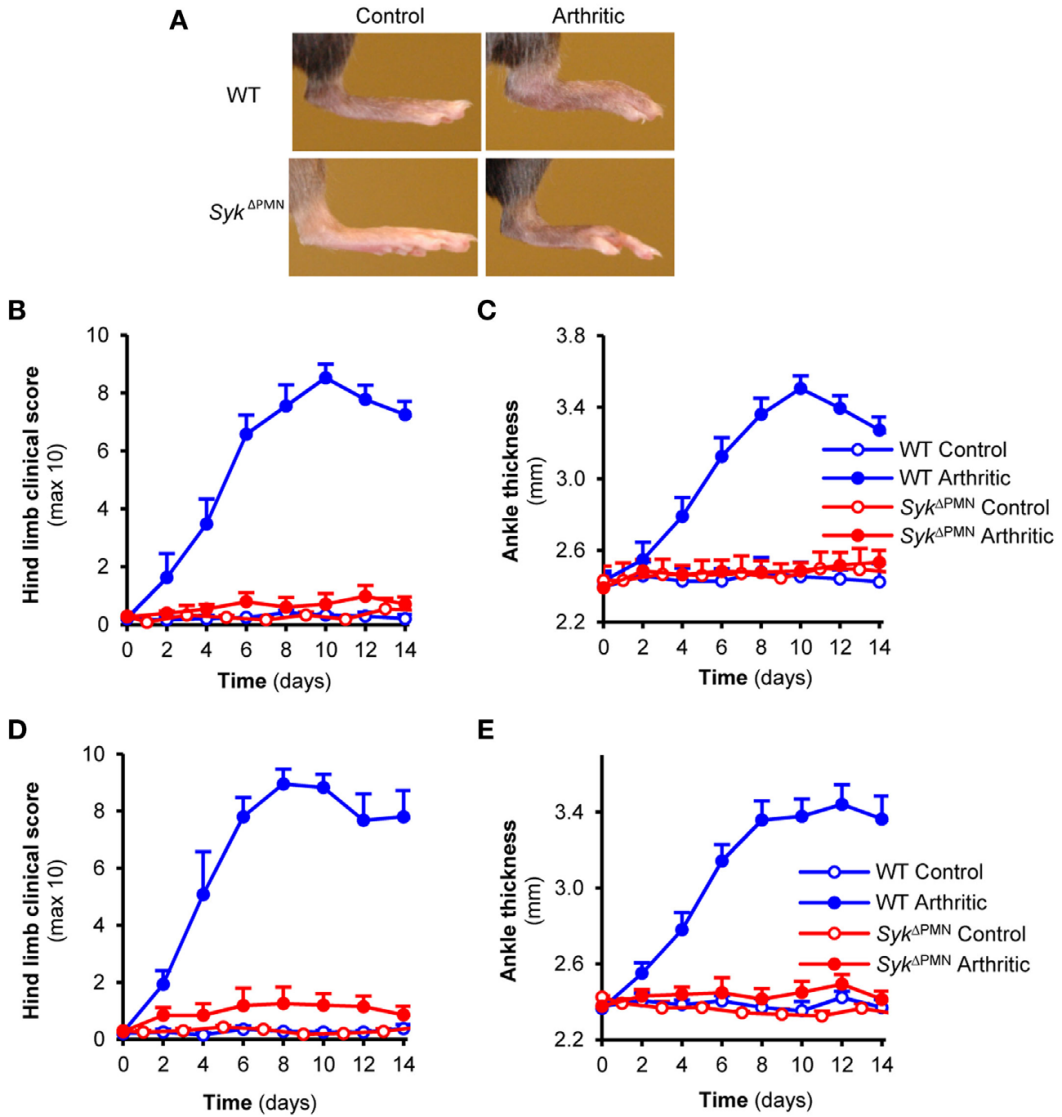
Neutrophil-specific Syk deletion attenuates experimental arthritis. Wild type (WT) and *Syk*^ΔPMN^ intact animals **(A–C)** or bone marrow chimeras **(D,E)** were injected with BxN (Control) or K/BxN (Arthritic) serum intraperitonally on day 0. Arthritis development was followed by photographing **(A)**, clinical scoring of the hind limbs **(B,D)**, and ankle thickness measurement **(C,E)**. Quantitative data show mean and SEM from three control and five to six arthritic serum-treated individual mice per group from three independent experiments **(B,C)** or from five control and five to seven arthritic serum-treated mice per group from three independent experiments **(D,E)**. See the text for actual *p* values.

We next tested the consequence of neutrophil-specific deletion of Syk on the development of K/BxN serum-transfer arthritis. As shown in Figure 4A, arthritogenic K/BxN serum triggered visible arthritis development in WT animals. However, no signs of arthritis could be observed in *Syk*^ΔPMN^ animals (Figure 4A). Quantitative kinetic analysis revealed that *Syk*^ΔPMN^ mice were nearly completely protected from development of clinical signs of arthritis (Figure 4B; *p* = 1.5 × 10^−5^) and arthritis-induced ankle swelling (Figure 4C; *p* = 1.7 × 10^−3^). Similar results could be observed when testing a larger cohort of bone marrow chimeras generated by transplanting WT or *Syk*^ΔPMN^ bone marrow cells into lethally irradiated WT recipients (Figures 4D,E; *p* = 6.2 × 10^−7^ and *p* = 3.2 × 10^−6^, respectively). Those results indicate a critical role for Syk expression within neutrophils for the development of autoantibody-induced arthritis *in vivo*.

### Normal Arthritis Development Upon Platelet-Specific Deletion of Syk

**Figure 5:**
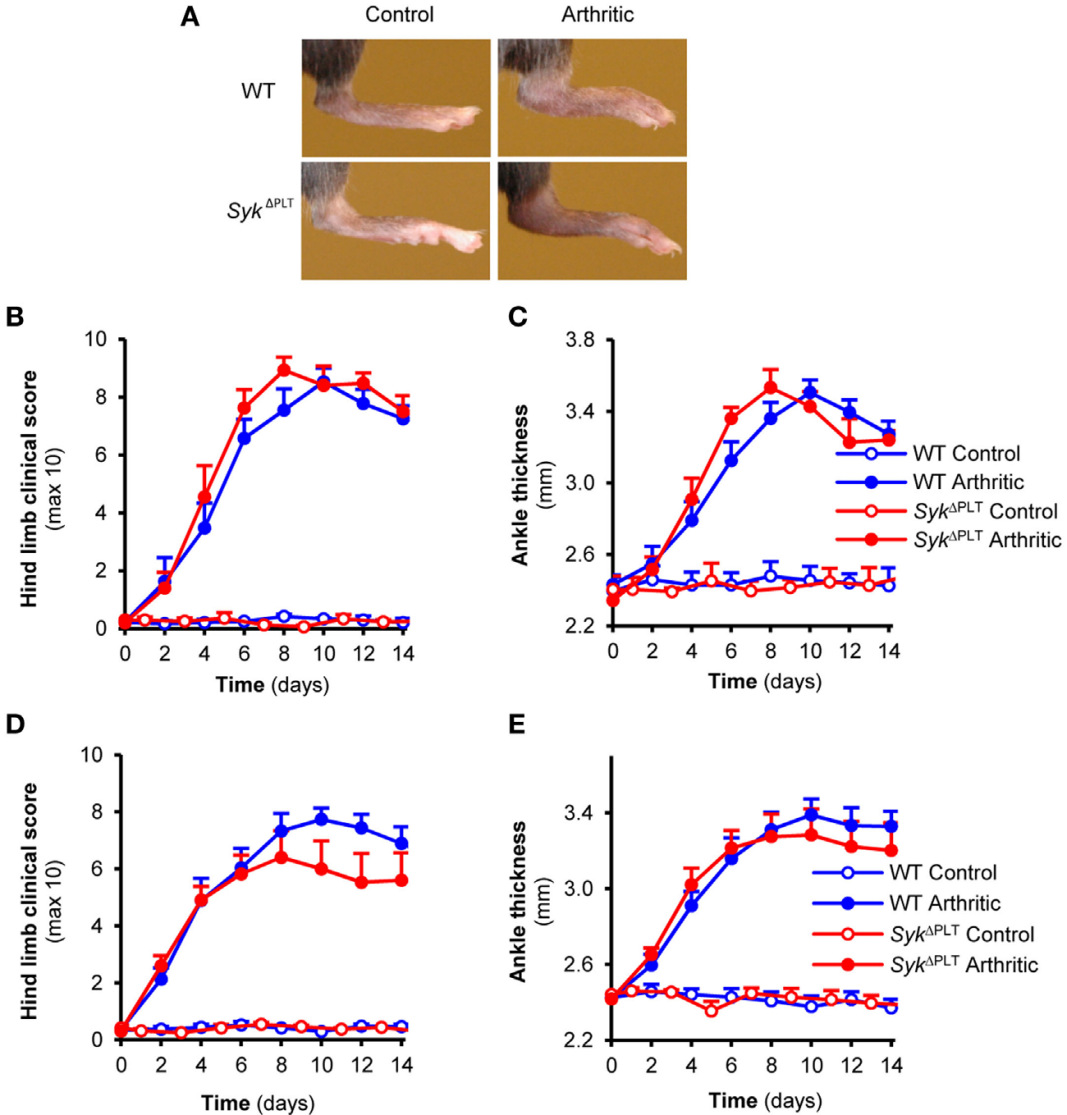
Platelet-specific Syk deletion has no effect on autoantibody-induced arthritis. Wild type (WT) and *Syk*^ΔPLT^ intact animals **(A–C)** or bone marrow chimeras **(D,E)** were injected with BxN (Control) or K/BxN (Arthritic) serum intraperitonally on day 0. Arthritis development was followed by photographing **(A)**, clinical scoring of the hind limbs **(B,D)**, and ankle thickness measurement **(C,E)**. Quantitative data show mean and SEM from three control and five arthritic serum-treated individual mice per group from three independent experiments **(B,C)** or from six control and eight to nine arthritic serum-treated mice per group from three independent experiments **(D,E)**. See the text for actual *p* values.

Boilard et al. previously showed that genetic deletion of the Syk-coupled GpVI collagen receptor of platelets strongly reduced arthritis development in the K/BxN serum-transfer model (10), suggesting an important role for Syk expression in platelets in this model (33). To test this hypothesis experimentally, we tested K/BxN serum-transfer arthritis in *Syk*^ΔPLT^ mice in which Syk was deleted in a platelet-specific manner. Contrary to our expectations, platelet-specific Syk deletion did not affect the development of visual signs of arthritis in our model (Figure 5A). Quantitative kinetic analysis did not reveal any effect of the *Syk*^ΔPLT^ mutation on the clinical appearance (Figure 5B; *p* = 0.51) or on the ankle thickness increase (Figure 5C; *p* = 0.76) either. Similar results were obtained when using bone marrow chimeras generated by transplanting WT or *Syk*^ΔPLT^ bone marrow cells into lethally irradiated WT recipients (Figures 5D,E; *p* = 0.49 and *p* = 0.9, respectively). Those results, together with the lack of Syk (Figure 2B) and the defective Syk-dependent functional activation (Figure 3B) of *Syk*^ΔPLT^ platelets indicate that Syk expression in platelets is not required for the development of K/BxN serum-transfer arthritis.

### Mast Cell-Specific Syk Deletion Does Not Affect Autoantibody-Induced Arthritis

**Figure 6:**
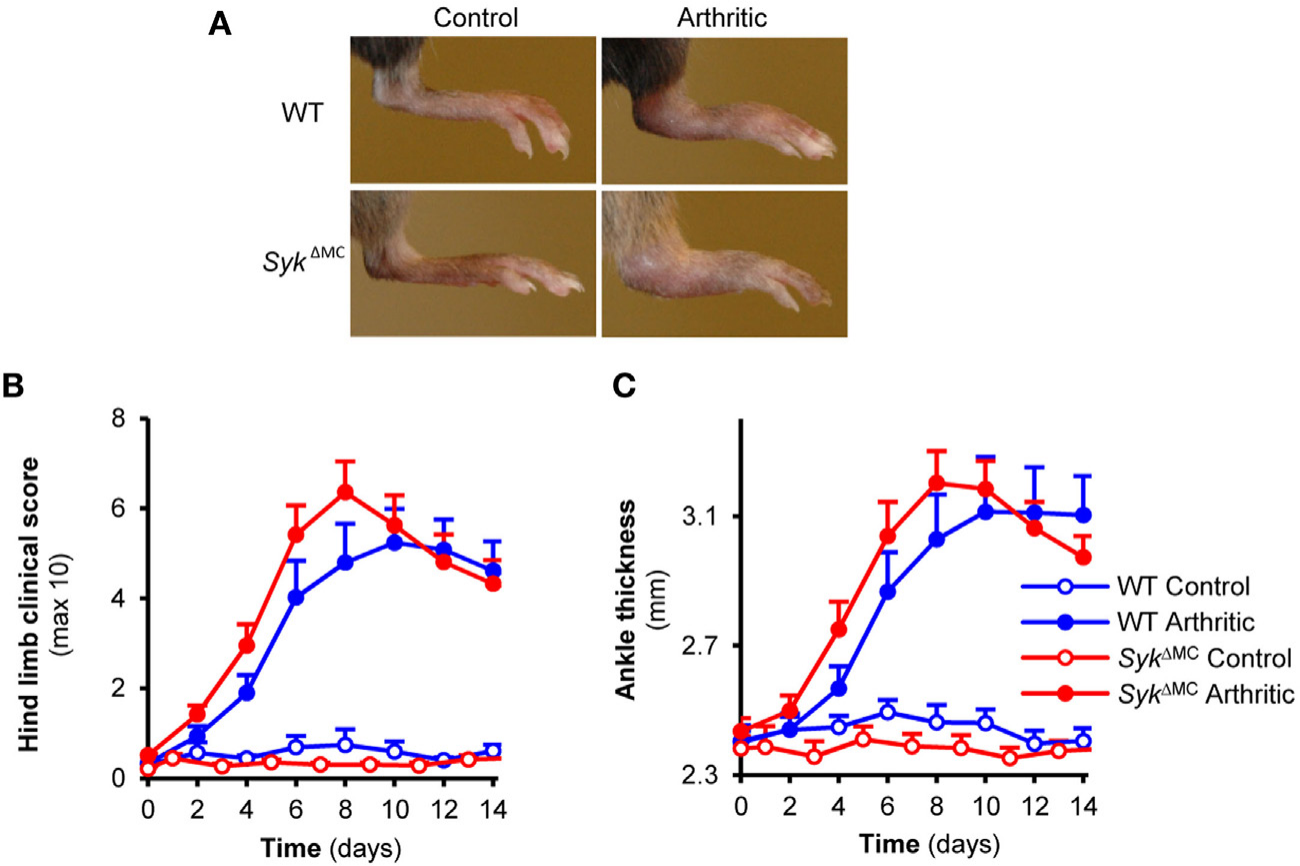
Mast cell-specific Syk deletion does not affect the effector phase of experimental arthritis. Wild type (WT) and *Syk*^ΔMC^ animals were injected with BxN (Control) or K/BxN (Arthritic) serum intraperitonally on day 0. Arthritis development was followed by photographing **(A)**, clinical scoring of the hind limbs **(B)**, and ankle thickness measurement **(C)**. Quantitative data show mean and SEM from seven to nine control and twelve to thirteen arthritic serum-treated individual mice per group from four independent experiments. See the text for actual *p* values.

Mast cells are one of the major targets of Syk function (14, 18) and they have also been proposed to participate in the development of K/BxN serum-transfer arthritis (9, 34). Therefore, we hypothesized that Syk expression in mast cells may be required for arthritis development in this model. To this end, we tested the development of K/BxN serum-transfer arthritis in *Syk*^ΔMC^ mice. As shown in Figure 6A, the *Syk*^ΔMC^ mutation did not affect the development of visible signs of arthritis in our model. Quantitative kinetic analysis did not reveal any inhibition of arthritis development either when scoring clinical signs of arthritis (Figure 6B; *p* = 0.38) or when measuring arthritis-induced increase of ankle thickness (Figure 6C; *p* = 0.37). By contrast, there was even a tendency of earlier arthritis development in the *Syk*^ΔMC^ animals (Figures 6B,C), raising the possibility of a negative role of Syk expressed in mast cells. Because of the radioresistance of mast cells, no bone marrow chimeras have been generated using *Syk*^ΔMC^ mice. The lack of inhibition of arthritis in *Syk*^ΔMC^ animals, together with the dramatic reduction of Syk expression (Figure 2C) and Syk-mediated functional responses (Figure 3C) in *Syk*^ΔMC^ mast cells indicates that Syk expression within mast cells is dispensable for arthritis development in the K/BxN serum-transfer model.

## Discussion

The Syk tyrosine kinase is critically involved in various inflammatory disease processes including the development of autoantibody-induced arthritis and dermatitis models (11, 17, 25). Given the wide expression of Syk in practically all hematopoietic lineages (11), understanding Syk function in a lineage-specific manner is of particular importance. Our results presented in this work indicate that of the three most prominent Syk-expressing lineages supposedly involved in the development of autoantibody-induced arthritis, Syk expression in neutrophils is critical, whereas that in platelets or mast cells is dispensable, for the development of K/BxN serum-transfer arthritis.

We and others have shown that Syk plays a critical role in various functional responses of neutrophils (16, 17, 19, 42, 43) without affecting neutrophil development (17, 19). Neutrophils have also been shown to be critical for the development of autoantibody-induced arthritis (6–8), likely at least in part through IgG IC-mediated activation of Fcγ-receptors expressed on the neutrophil cell surface (29), as well as by yet incompletely understood neutrophil-mediated initial vascular changes (8). Based on those studies, we hypothesized that Syk expression within neutrophils is critical for autoantibody-induced arthritis development. Our results confirmed that hypothesis, and they were also in line with prior studies from other groups (32) and our own analysis of neutrophil-specific deletion of the CARD9 adapter protein, a supposedly downstream effector of Syk (31). Though it is at present incompletely understood how Syk within neutrophils participates in autoantibody-induced arthritis development, our prior studies showing defective release of proinflammatory mediators by Syk-deficient neutrophils despite normal intrinsic migratory capacity of the cells (17, 19, 31) suggest that Syk, similar to Src-family kinases (29), participates in the amplification of neutrophil recruitment by neutrophil-derived proinflammatory mediators (44).

In contrast to our neutrophil-specific deletion studies, our platelet-specific deletion experiments did not support our hypothesis based on literature data. Though Syk is not required for platelet development (20), it plays a critical role in various platelet functions (11) including α_IIb_β_3_ integrin-dependent platelet spreading (20), responses mediated by the hemITAM-coupled C-type lectin CLEC-2 (45), as well as signaling downstream of GpVI, an ITAM-coupled collagen-receptor of platelets (40, 41). GpVI is closely related to Fcα-receptors and it is directly associated with, and supposedly signals through, the ITAM-containing Fc-receptor γ-chain (FcRγ) (40, 41, 46–49). Platelets and, specifically, GpVI has been shown to play a critical role in the development of K/BxN serum-transfer arthritis (10), suggesting that Syk expression downstream of platelet GpVI is critically involved in arthritis development in this model (33). Our results of normal arthritis development upon platelet-specific deletion of Syk (Figure 5) despite practically complete lack of Syk from platelets (Figure 2) and completely defective GpVI-mediated *in vitro* platelet function (Figure 3) argues against that hypothesis. There are several possible explanations for those findings. Though GpVI is associated with the ITAM-containing FcRγ adapter, it may be able to bypass the ITAM-Syk pathway under certain conditions, using FcRγ as a chaperone required for cell surface expression but not as an ITAM-mediated signaling adaptor. Platelets have also been proposed to interact with fibroblast-like synoviocytes in a COX-1-dependent manner which is independent of platelet GpVI or FcRγ expression (50). This pathway may be able to compensate for the defective GpVI–FcRγ–Syk pathway upon platelet-specific Syk deletion. We also cannot exclude the possibility that GpVI needs to be expressed in a non-platelet lineage to support autoantibody-induced arthritis in mice. Finally, technical details such as a role for the small remaining Syk expression after Cre-mediated *Syk* deletion, or different experimental conditions may also account for the different conclusions drawn from our study and from those proposing a critical role for the platelet GpVI–FcRγ–Syk pathway in autoantibody-induced arthritis (10, 33). It should also be mentioned that our study focused on visible signs of arthritis and therefore we cannot exclude the possibility that Syk expression in platelets modulates the inflammation process by a mechanism not clearly visible by macroscopic inspection.

In the third part of our study, we tested the role of Syk in mast cells during autoantibody-induced arthritis. Syk has been shown to play a critical role in mast cell function without affecting mast cell survival (14, 18) and mast cells were proposed to be important players in autoantibody-induced arthritis development (9). Therefore, we hypothesized that Syk expression in mast cells may play a role in the development of K/BxN serum-transfer arthritis. Our results showing normal arthritis development in that model upon mast cell-specific Syk deletion (Figure 6) despite strongly reduced Syk expression (Figure 2) and defective Syk-mediated functional responses (Figure 3) in mast cells argue against that possibility. There are several possible explanations for those findings. Since the mechanism of how mast cells contribute to IgG autoantibody-induced disease pathogenesis is incompletely understood, it is possible that mast cells use a Syk-independent signal transduction pathway during K/BxN serum-transfer arthritis (e.g., when mast cells are not directly activated by the autoantibody-containing ICs, but rather indirectly through Syk-independent chemokine, cytokine, or PRR pathways). It should also be mentioned that follow-up studies have questioned the critical role of mast cells in autoantibody-induced arthritis development (51, 52), pointing to difficulties of the interpretation of data obtained with different mast cell-deficient mouse strains. Indeed, our limited preliminary studies also suggested that the role of mast cells is highly dependent on the experimental conditions used for triggering autoantibody-induced arthritis in mice (Z. Jakus and A. M., unpublished observations). Finally, given that our experiments focused on visible signs of inflammation, we cannot exclude the possibility that Syk expression in mast cells may modulate arthritis development or the overall inflammation process in a manner not clearly visible by macroscopic assessment.

Besides neutrophils, platelets, and mast cells, Syk is also expressed in other lineages possibly involved in arthritis development. B-cells are one of the most prominent lineages requiring Syk function (12, 13). However, it is unlikely that Syk expressed in B-cells contributes to K/BxN serum-transfer arthritis since that model mimics the post-immunization effector phase of autoimmune arthritis and it develops normally even in the absence of B-cells in μMT-deficient or Rag-deficient mice (3). Macrophages have been proposed to be important players in the development of K/BxN serum-transfer arthritis (53). Unfortunately, currently available techniques do not allow the proper analysis of the *in vivo* relevance of Syk expression within macrophages because of the limited spectrum/specificity of the available macrophage-specific Cre-expressing mouse strains (54). We have previously shown that Syk is critically involved in osteoclast development and function (23). Though understanding the role of Syk in arthritis-induced bone erosions would be of clear importance, this question is beyond the scope of the present study focusing on the inflammatory aspect of autoantibody-induced disease processes.

Taken together, our results provide understanding of the role of Syk in autoantibody-induced arthritis at the cellular lineage level. Our findings indicate a critical role for Syk expression in neutrophils, but refute prior assumptions for the role of Syk in platelets and argue against a role for Syk expression in mast cells. Those results will strongly contribute to the understanding of the pathomechanism of autoantibody-mediated disease processes at the cellular and molecular level.

## Ethics Statement

All animal experiments were approved by the Animal Experimentation Review Board of the Semmelweis University.

## Author Contributions

TN and AM conceived the study, designed the experiments, and wrote the manuscript. TN, KF, KS, OV, and LK-P performed the experiments. TN, KF, and AM analyzed and interpreted the data. AM supervised the project.

## Conflict of Interest Statement

The authors declare that the research was conducted in the absence of any commercial or financial relationships that could be construed as a potential conflict of interest. The reviewer KB and handling editor declared their shared affiliation.

## Supplementary Material

Figure S1 Flow cytometric analysis of bone marrow chimeras and Western blot images with more details. **(A)** Flow cytometric analysis of CD45.2-positive donor-derived neutrophils in the peripheral blood of wild type (WT) and *Syk*^ΔPMN^ bone marrow chimeras 4 weeks after bone marrow transplantation. **(B–D)** Detailed Western blot images showing the expression of Syk in neutrophil **(B)**, platelet **(C)**, or mast cell **(D)** lysates from Figure 1D.

Figure S2 Detailed Western blot images showing the efficacy and specificity of lineage-specific Syk deletion in whole cell lysates of neutrophils **(A)**, platelets **(B)**, and mast cells **(C)** from Figure 2.

## References

[1] Suurmond J Diamond B. Autoantibodies in systemic autoimmune diseases: specificity and pathogenicity. J Clin Invest (2015) 125:2194–202. 10.1172/JCI78084 25938780 PMC4497746

[2] Kouskoff V Korganow AS Duchatelle V Degott C Benoist C Mathis D. Organ-specific disease provoked by systemic autoimmunity. Cell (1996) 87:811–22. 10.1016/S0092-8674(00)81989-3 8945509

[3] Korganow AS Ji H Mangialaio S Duchatelle V Pelanda R Martin T From systemic T cell self-reactivity to organ-specific autoimmune disease via immunoglobulins. Immunity (1999) 10:451–61. 10.1016/S1074-7613(00)80045-X 10229188

[4] Matsumoto I Staub A Benoist C Mathis D. Arthritis provoked by linked T and B cell recognition of a glycolytic enzyme. Science (1999) 286:1732–5. 10.1126/science.286.5445.1732 10576739

[5] Ji H Ohmura K Mahmood U Lee DM Hofhuis FM Boackle SA Arthritis critically dependent on innate immune system players. Immunity (2002) 16:157–68. 10.1016/S1074-7613(02)00275-3 11869678

[6] Wipke BT Allen PM. Essential role of neutrophils in the initiation and progression of a murine model of rheumatoid arthritis. J Immunol (2001) 167:1601–8. 10.4049/jimmunol.167.3.1601 11466382

[7] Jonsson H Allen P Peng SL. Inflammatory arthritis requires Foxo3a to prevent Fas ligand-induced neutrophil apoptosis. Nat Med (2005) 11:666–71. 10.1038/nm1248 15895074

[8] Binstadt BA Patel PR Alencar H Nigrovic PA Lee DM Mahmood U Particularities of the vasculature can promote the organ specificity of autoimmune attack. Nat Immunol (2006) 7:284–92. 10.1038/ni1306 16444258

[9] Lee DM Friend DS Gurish MF Benoist C Mathis D Brenner MB. Mast cells: a cellular link between autoantibodies and inflammatory arthritis. Science (2002) 297:1689–92. 10.1126/science.1073176 12215644

[10] Boilard E Nigrovic PA Larabee K Watts GF Coblyn JS Weinblatt ME Platelets amplify inflammation in arthritis via collagen-dependent microparticle production. Science (2010) 327:580–3. 10.1126/science.1181928 20110505 PMC2927861

[11] Mócsai A Ruland J Tybulewicz VL. The SYK tyrosine kinase: a crucial player in diverse biological functions. Nat Rev Immunol (2010) 10:387–402. 10.1038/nri2765 20467426 PMC4782221

[12] Turner M Mee PJ Costello PS Williams O Price AA Duddy LP Perinatal lethality and blocked B-cell development in mice lacking the tyrosine kinase Syk. Nature (1995) 378:298–302. 10.1038/378298a0 7477352

[13] Cheng AM Rowley B Pao W Hayday A Bolen JB Pawson T. Syk tyrosine kinase required for mouse viability and B-cell development. Nature (1995) 378:303–6. 10.1038/378303a0 7477353

[14] Costello PS Turner M Walters AE Cunningham CN Bauer PH Downward J Critical role for the tyrosine kinase Syk in signalling through the high affinity IgE receptor of mast cells. Oncogene (1996) 13:2595–605. 9000133

[15] Crowley MT Costello PS Fitzer-Attas CJ Turner M Meng F Lowell C A critical role for Syk in signal transduction and phagocytosis mediated by Fcγ receptors on macrophages. J Exp Med (1997) 186:1027–39. 10.1084/jem.186.7.1027 9314552 PMC2199061

[16] Kiefer F Brumell J Al-Alawi N Latour S Cheng A Veillette A The Syk protein tyrosine kinase is essential for Fcγ receptor signaling in macrophages and neutrophils. Mol Cell Biol (1998) 18:4209–20. 10.1128/MCB.18.7.4209 9632805 PMC109005

[17] Németh T Virtic O Sitaru C Mócsai A The Syk tyrosine kinase is required for skin inflammation in an in vivo mouse model of epidermolysis bullosa acquisita. J Invest Dermatol (2017) 137:2131–9. 10.1016/j.jid.2017.05.017 28576735 PMC5624865

[18] Mócsai A Zhang H Jakus Z Kitaura J Kawakami T Lowell CA. G-protein-coupled receptor signaling in Syk-deficient neutrophils and mast cells. Blood (2003) 101:4155–63. 10.1182/blood-2002-07-2346 12531806

[19] Mócsai A Zhou M Meng F Tybulewicz VL Lowell CA. Syk is required for integrin signaling in neutrophils. Immunity (2002) 16:547–58. 10.1016/S1074-7613(02)00303-5 11970878

[20] Obergfell A Eto K Mócsai A Buensuceso C Moores SL Brugge JS Coordinate interactions of Csk, Src, and Syk kinases with αIIbβ3 initiate integrin signaling to the cytoskeleton. J Cell Biol (2002) 157:265–75. 10.1083/jcb.200112113 11940607 PMC2199242

[21] Jakus Z Fodor S Abram CL Lowell CA Mócsai A Immunoreceptor-like signaling by β2 and β3 integrins. Trends Cell Biol (2007) 17:493–501. 10.1016/j.tcb.2007.09.001 17913496

[22] Werninghaus K Babiak A Gross O Holscher C Dietrich H Agger EM Adjuvanticity of a synthetic cord factor analogue for subunit Mycobacterium tuberculosis vaccination requires FcRγ-Syk-Card9-dependent innate immune activation. J Exp Med (2009) 206:89–97. 10.1084/jem.20081445 19139169 PMC2626670

[23] Mócsai A Humphrey MB Van Ziffle JA Hu Y Burghardt A Spusta SC The immunomodulatory adapter proteins DAP12 and Fc receptor γ-chain (FcRγ) regulate development of functional osteoclasts through the Syk tyrosine kinase. Proc Natl Acad Sci U S A (2004) 101:6158–63. 10.1073/pnas.0401602101 15073337 PMC395939

[24] Hirahashi J Mekala D Van Ziffle J Xiao L Saffaripour S Wagner DD Mac-1 signaling via Src-family and Syk kinases results in elastase-dependent thrombohemorrhagic vasculopathy. Immunity (2006) 25:271–83. 10.1016/j.immuni.2006.05.014 16872848

[25] Jakus Z Simon E Balázs B Mócsai A. Genetic deficiency of Syk protects mice from autoantibody-induced arthritis. Arthritis Rheum (2010) 62:1899–910. 10.1002/art.27438 20201079 PMC2972644

[26] Gross O Poeck H Bscheider M Dostert C Hannesschlager N Endres S Syk kinase signalling couples to the Nlrp3 inflammasome for anti-fungal host defence. Nature (2009) 459:433–6. 10.1038/nature07965 19339971

[27] Abtahian F Guerriero A Sebzda E Lu MM Zhou R Mócsai A Regulation of blood and lymphatic vascular separation by signaling proteins SLP-76 and Syk. Science (2003) 299:247–51. 10.1126/science.1079477 12522250 PMC2982679

[28] Geahlen RL. Getting Syk: spleen tyrosine kinase as a therapeutic target. Trends Pharmacol Sci (2014) 35:414–22. 10.1016/j.tips.2014.05.007 24975478 PMC4119858

[29] Kovács M Németh T Jakus Z Sitaru C Simon E Futosi K The Src family kinases Hck, Fgr, and Lyn are critical for the generation of the in vivo inflammatory environment without a direct role in leukocyte recruitment. J Exp Med (2014) 211:1993–2011. 10.1084/jem.20132496 25225462 PMC4172222

[30] Jakus Z Simon E Frommhold D Sperandio M Mócsai A Critical role of phospholipase Cγ2 in integrin and Fc receptor-mediated neutrophil functions and the effector phase of autoimmune arthritis. J Exp Med (2009) 206:577–93. 10.1084/jem.20081859 19273622 PMC2699137

[31] Németh T Futosi K Sitaru C Ruland J Mócsai A. Neutrophil-specific deletion of the CARD9 gene expression regulator suppresses autoantibody-induced inflammation in vivo. Nat Commun (2016) 7:11004. 10.1038/ncomms11004 27032818 PMC4821996

[32] Elliott ER Van Ziffle JA Scapini P Sullivan BM Locksley RM Lowell CA. Deletion of Syk in neutrophils prevents immune complex arthritis. J Immunol (2011) 187:4319–30. 10.4049/jimmunol.1100341 21918195 PMC3186826

[33] Boilard E Blanco P Nigrovic PA. Platelets: active players in the pathogenesis of arthritis and SLE. Nat Rev Rheumatol (2012) 8:534–42. 10.1038/nrrheum.2012.118 22868927

[34] Nigrovic PA Binstadt BA Monach PA Johnsen A Gurish M Iwakura Y Mast cells contribute to initiation of autoantibody-mediated arthritis via IL-1. Proc Natl Acad Sci U S A (2007) 104:2325–30. 10.1073/pnas.0610852103 17277081 PMC1892913

[35] Passegue E Wagner EF Weissman IL. JunB deficiency leads to a myeloproliferative disorder arising from hematopoietic stem cells. Cell (2004) 119:431–43. 10.1016/j.cell.2004.10.010 15507213

[36] Tiedt R Schomber T Hao-Shen H Skoda RC. Pf4-Cre transgenic mice allow the generation of lineage-restricted gene knockouts for studying megakaryocyte and platelet function in vivo. Blood (2007) 109:1503–6. 10.1182/blood-2006-04-020362 17032923

[37] Scholten J Hartmann K Gerbaulet A Krieg T Muller W Testa G Mast cell-specific Cre/loxP-mediated recombination in vivo. Transgenic Res (2008) 17:307–15. 10.1007/s11248-007-9153-4 17972156 PMC2268725

[38] Saijo K Schmedt C Su IH Karasuyama H Lowell CA Reth M Essential role of Src-family protein tyrosine kinases in NF-κB activation during B cell development. Nat Immunol (2003) 4:274–9. 10.1038/ni893 12563261

[39] Németh T Futosi K Hably C Brouns MR Jakob SM Kovács M Neutrophil functions and autoimmune arthritis in the absence of p190RhoGAP: generation and analysis of a novel null mutation in mice. J Immunol (2010) 185:3064–75. 10.4049/jimmunol.0904163 20675588 PMC3064944

[40] Meinders M Hoogenboezem M Scheenstra MR De Cuyper IM Papadopoulos P Németh T Repercussion of megakaryocyte-specific Gata1 loss on megakaryopoiesis and the hematopoietic precursor compartment. PLoS One (2016) 11:e0154342. 10.1371/journal.pone.0154342 27152938 PMC4859556

[41] Watson SP Herbert JM Pollitt AY. GPVI and CLEC-2 in hemostasis and vascular integrity. J Thromb Haemost (2010) 8:1456–67. 10.1111/j.1538-7836.2010.03875.x 20345705

[42] Schymeinsky J Sindrilaru A Frommhold D Sperandio M Gerstl R Then C The Vav binding site of the non-receptor tyrosine kinase Syk at Tyr 348 is critical for β2 integrin (CD11/CD18)-mediated neutrophil migration. Blood (2006) 108:3919–27. 10.1182/blood-2005-12-030387 16882714

[43] Frommhold D Mannigel I Schymeinsky J Mócsai A Poeschl J Walzog B Spleen tyrosine kinase Syk is critical for sustained leukocyte adhesion during inflammation in vivo. BMC Immunol (2007) 8:31. 10.1186/1471-2172-8-31 18045459 PMC2217554

[44] Németh T Mócsai A. Feedback amplification of neutrophil function. Trends Immunol (2016) 37:412–24. 10.1016/j.it.2016.04.002 27157638

[45] Suzuki-Inoue K Fuller GL Garcia A Eble JA Pohlmann S Inoue O A novel Syk-dependent mechanism of platelet activation by the C-type lectin receptor CLEC-2. Blood (2006) 107:542–9. 10.1182/blood-2005-05-1994 16174766

[46] Gibbins JM Okuma M Farndale R Barnes M Watson SP Glycoprotein VI is the collagen receptor in platelets which underlies tyrosine phosphorylation of the Fc receptor γ-chain. FEBS Lett (1997) 413:255–9. 10.1016/S0014-5793(97)00926-5 9280292

[47] Tsuji M Ezumi Y Arai M Takayama H A novel association of Fc receptor γ-chain with glycoprotein VI and their co-expression as a collagen receptor in human platelets. J Biol Chem (1997) 272:23528–31. 10.1074/jbc.272.38.23528 9295288

[48] Clemetson JM Polgar J Magnenat E Wells TN Clemetson KJ The platelet collagen receptor glycoprotein VI is a member of the immunoglobulin superfamily closely related to FcαR and the natural killer receptors. J Biol Chem (1999) 274:29019–24. 10.1074/jbc.274.41.29019 10506151

[49] Kasirer-Friede A Kahn ML Shattil SJ. Platelet integrins and immunoreceptors. Immunol Rev (2007) 218:247–64. 10.1111/j.1600-065X.2007.00532.x 17624957

[50] Boilard E Larabee K Shnayder R Jacobs K Farndale RW Ware J Platelets participate in synovitis via Cox-1-dependent synthesis of prostacyclin independently of microparticle generation. J Immunol (2011) 186:4361–6. 10.4049/jimmunol.1002857 21357261 PMC3208424

[51] Zhou JS Xing W Friend DS Austen KF Katz HR Mast cell deficiency in KitW-sh mice does not impair antibody-mediated arthritis. J Exp Med (2007) 204:2797–802. 10.1084/jem.20071391 17998392 PMC2118523

[52] Feyerabend TB Weiser A Tietz A Stassen M Harris N Kopf M Cre-mediated cell ablation contests mast cell contribution in models of antibody- and T cell-mediated autoimmunity. Immunity (2011) 35:832–44. 10.1016/j.immuni.2011.09.015 22101159

[53] Solomon S Rajasekaran N Jeisy-Walder E Snapper SB Illges H. A crucial role for macrophages in the pathology of K/BxN serum-induced arthritis.Eur J Immunol (2005) 35:3064–73. 10.1002/eji.200526167 16180250

[54] Abram CL Roberge GL Hu Y Lowell CA. Comparative analysis of the efficiency and specificity of myeloid-Cre deleting strains using ROSA-EYFP reporter mice. J Immunol Methods (2014) 408:89–100. 10.1016/j.jim.2014.05.009 24857755 PMC4105345

