# Supplementary figures and images for: Lineage-Specific Analysis of Syk Function in Autoantibody-Induced Arthritis
Source: Front Immunol. 2018 Mar 19;9:555. doi: 10.3389/fimmu.2018.00555 (PMC5867294; doi:10.3389/fimmu.2018.00555)

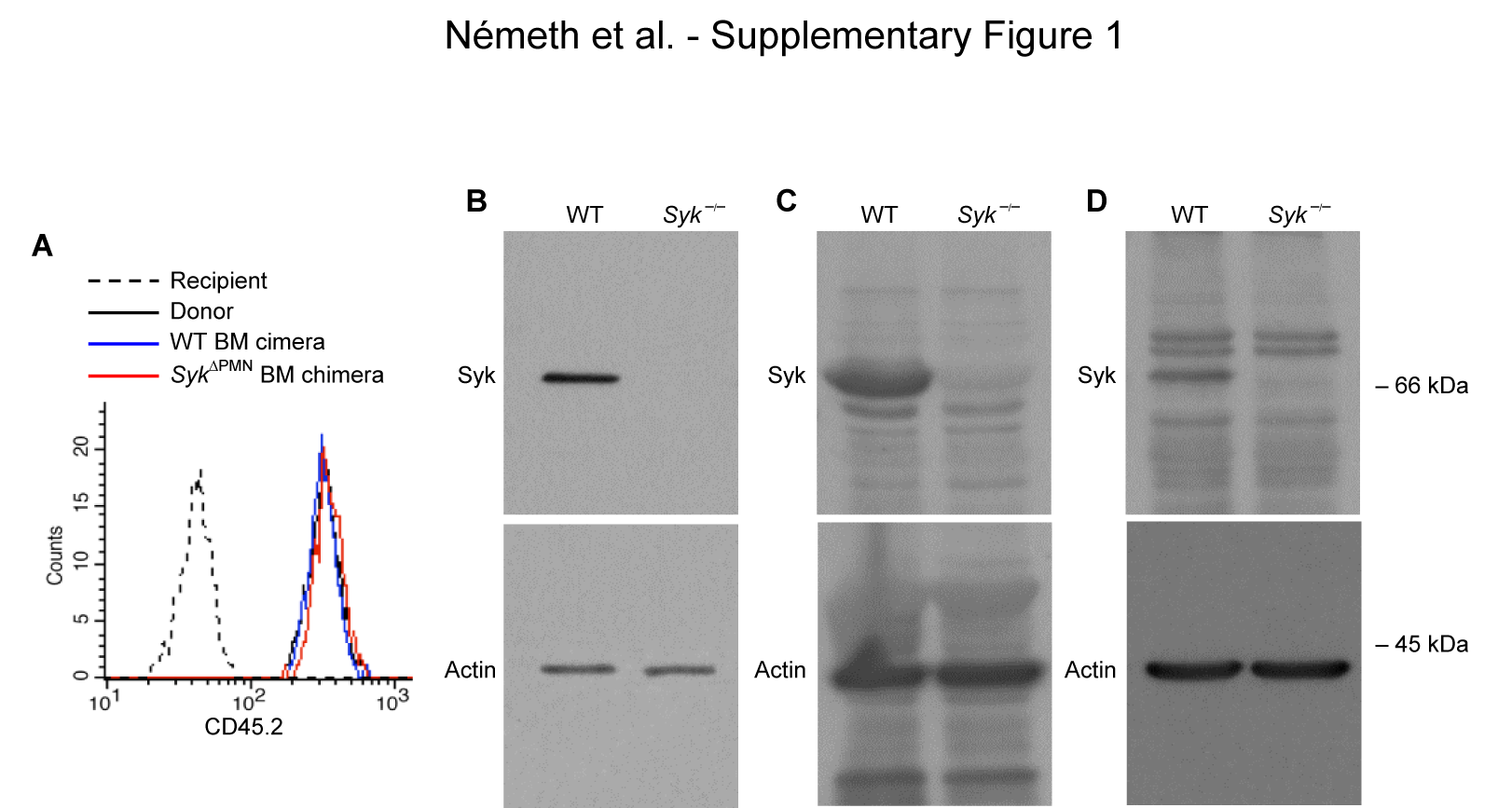

Supplement: Figure S1 — Flow cytometric analysis of bone marrow chimeras and Western blot images with more details. (A) Flow cytometric analysis of CD45.2-positive donor-derived neutrophils in the peripheral blood of wild type (WT) and SykΔPMN bone marrow chimeras 4 weeks after bone marrow transplantation. (B–D) Detailed Western blot images showing the expression of Syk in neutrophil (B), platelet (C), or mast cell (D) lysates from Figure 1D. [file Image_1.tif]

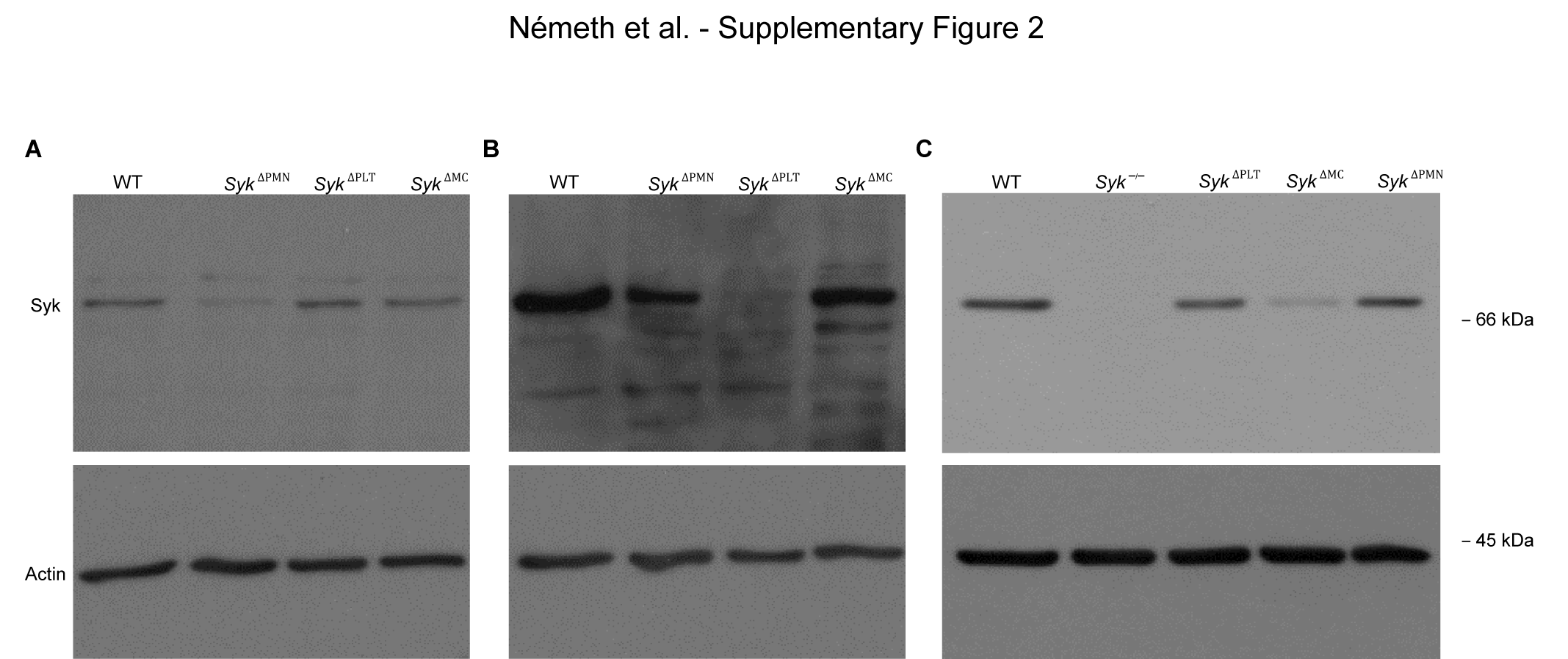

Supplement: Figure S2 — Detailed Western blot images showing the efficacy and specificity of lineage-specific Syk deletion in whole cell lysates of neutrophils (A), platelets (B), and mast cells (C) from Figure 2. [file Image_2.tif]
